# Genetics of atrioventricular canal defects

**DOI:** 10.1186/s13052-020-00825-4

**Published:** 2020-05-13

**Authors:** Flaminia Pugnaloni, Maria Cristina Digilio, Carolina Putotto, Enrica De Luca, Bruno Marino, Paolo Versacci

**Affiliations:** 1 Department of Pediatrics, Obstetrics and Gynecology, “Sapienza” University of Rome, Policlinico Umberto I, Viale Regina Elena, 324, 00161 Rome, Italy; 2 Medical Genetics Unit, Bambino Gesù Children’s Hospital and Research Institute, 00165 Rome, Italy

**Keywords:** Congenital heart disease, Atrioventricular canal defect, Genetics

## Abstract

Atrioventricular canal defect (AVCD) represents a quite common congenital heart defect (CHD) accounting for 7.4% of all cardiac malformations. AVCD is a very heterogeneous malformation that can occur as a phenotypical cardiac aspect in the context of different genetic syndromes but also as an isolated, non-syndromic cardiac defect. AVCD has also been described in several pedigrees suggesting a pattern of familiar recurrence. Targeted Next Generation Sequencing (NGS) techniques are proved to be a powerful tool to establish the molecular heterogeneity of AVCD.

Given the complexity of cardiac embryology, it is not surprising that multiple genes deeply implicated in cardiogenesis have been described mutated in patients with AVCD. This review attempts to examine the recent advances in understanding the molecular basis of this complex CHD in the setting of genetic syndromes or in non-syndromic patients.

## Introduction

The atrioventricular canal defect (AVCD), also called atrioventricular septal defect, is a quite common congenital heart defect (CHD), accounting for 7.4% of all cardiac malformations. It can be anatomically classified in complete, partial and intermediate types. Complete AVCD includes ostium primum atrial septal defect, a common atrioventricular valve and a confluent posterior ventricular septal defect located in the inlet portion of ventricular septum. Partial AVCD is characterized by ostium primun septal defect and two distinct orifices of the atrioventricular valves with cleft of the antero-medial leaflet of the mitral valve. The intermediate AVCD has a restrictive ventricular septal defect associated with anatomical characteristics of partial AVCD [1].

From an embryological point of view, AVCD was traditionally considered caused by a primary intracardiac mechanism consisting in the maldevelopment of atrioventricular endocardial cushions in relation to defects of extracellular matrix, leading to absent or incomplete fusion of ventral (antero-superior) and dorsal (postero-inferior) atrioventricular cushions [2–4]. Nevertheless, the hypothesis that extracardiac progenitor cells contribute also to the growth of the inlet part of the heart has been postulated following the experimental studies in chick embryos performed by Maria Victoria de la Cruz from 1977 on. In fact, later studies have confirmed that a population of extramesenchymal cells known as *spina vestibuli* or *dorsal mesenchymal protrusion (DMP)*, arising from the posterior segment of the secondary heart field (SHF) in the splanchnic mesoderm, grow towards the atrial surface of the primitive atrioventricular canal, in particular towards the inferior dorsal endocardial cushion, to close the primary atrial foramen and form the atrioventricular junction [5–9].

**Fig. 1:**
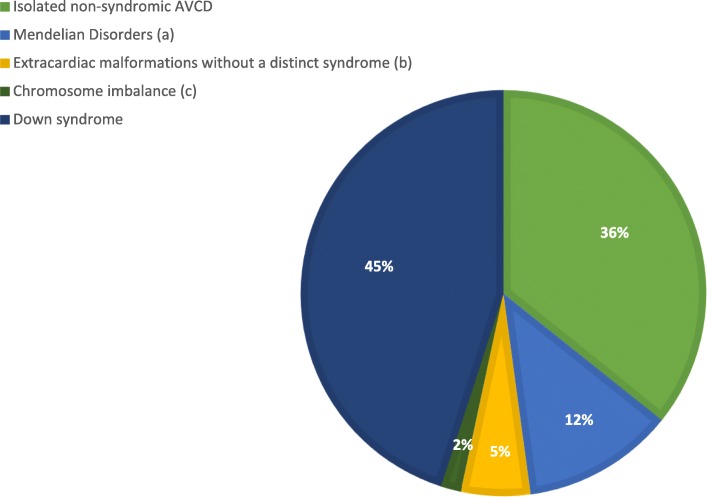
Distribution of AVCD with and without Down syndome modified by Digilio, M.C.; Marino, B.;Toscano, A.; Giannotti, A.; Dallapiccola, B. Atrioventricular canal defect without Down syndrome: a heterogeneous malformation. Am J Med Genet. 1999 Jul 16;85(2):140–6. (**a**) Mendelian Disorders: Noonan, Ellis-van Crevels, VACTERL, Oro-facio-digital II, Smith-Lemli-Opitz, DiGeorge,Bardet-Biedl, CHARGE. (**b**) Extracardiac malformations: Facial anomalies,dental anomalies, skeletal anomalies,gastrointestinal anomalies, glaucoma, mental retardation, (**c**) Chromosome imbalance: del 8 p21-pter; del 8 p23-pter; del 8 p21-p23;del4 q31-q32; 47, XX, + 18; 47, XY,+ 9;45,X

The AVCD is associated with extracardiac defects in about 75% of the cases and presents strong genetic association [10–13]. The best known genetic syndrome associated with AVCD is Down syndrome (DS) (45% of the cases) [10–13]. Other chromosomal or monogenic syndromes are accounting for about 15% of the cases [13]. Moreover, AVCD is associated with *heterotaxy* in additional 15% of the cases. Isolated, non-syndromic AVCD accounts for a percentage of about 36%. It is notable that among non-syndromic cases, a percentage of about 3.5% show a familial pattern of recurrence (Fig. 1).

It is noteworthy that AVCD displays anatomic variability possibly related to different and distinct genetic causes. Nevertheless, a common point seems to be causally implicated in several disorders linked to AVCD. In fact, clinical and molecular studies have demonstrated that several disease genes implicated in syndromes with AVCD encode proteins that participate in ciliary function. This was in agreement with previously known observation that dysfunction of the nodal cilium can result in left-right axis defects in vertebrates [14, 15]. Dysfunction in cilia can lead to several human genetic disorders with overlapping phenotypes, the so called “ciliopathies” [16, 17]. The ciliary membranes harbor receptors for crucial signaling cascades, including Hedgehog signaling [18, 19]. A link between AVCD and cilia abnormalities through a specific pathogenetic pathway involving Hedgehog signaling has been recognized in several syndromes with AVCD [20–23].

### Syndromic AVCD and chromosomal anomalies

**Down syndrome** is the most frequent genetic condition associated with AVCD. CHDs are diagnosed in 40–50% of these patients [24]. In this syndrome AVCD is frequently complete, showing a “simple type”, since rarely associated with other cardiac anomalies, with the exception of tetralogy of Fallot [25, 26]. In particular, left-sided obstructions are significantly more rare in patients with DS and AVCD in comparison with patients with AVCD and normal chromosomes [11, 24, 27]. Clinical studies on surgical prognosis of AVCD have shown that corrective surgery in patients with DS results in lower mortality and morbidity rates, compared to the children without trisomy 21 [28, 29].

From the molecular point of view, several genes located in the “CHD critical region” on chromosome 21 have been long investigated as a cause of AVCD, including *DSCAM*, *COL6A1*, *COL6A2*, and *DSCR1* [30, 31]. Additional genes mapping on different chromosomes including *CRELD1*, *FBLN2*, *FRZB*, and *GATA5* have been studied [32]*.* Particularly, the interaction between trisomic genes and modifiers on different chromosomes has been supported in experimental studies using mouse models of DS with high prevalence of CHD, in which loss-of-function alleles of *Creld1* or *Hey2* genes have been crossed with the trisomic background [33]. In addition, mouse models have evidenced the involvement of the Shh signaling pathway also in DS, since it has demonstrated that cerebral, skin, liver and intestine mice trisomic cells have a defective mitogenic Shh activity with cell proliferation impairment due to a higher expression of *Ptch1*, a receptor normally repressing the Shh pathway, located on Cr9 [34].

### Deletion 8p23

Deletion of the terminal part of the short arm of chromosome 8 (del 8p23) is the second chromosomal anomaly associated with AVCD [13]. Cardiac malformations are diagnosed in two third of the patients and AVCD is detected in about 40% of the cases [35]. AVCD is generally complete, with a frequent association with pulmonary valve stenosis and Tetralogy of Fallot [36, 37]. Heart defects as dextrocardia, abnormalities of the pulmonary and systemic venous returns, common atrium, single ventricle and transposition of the great arteries are also found in a group of patients with del 8p23 [35]. Some of these malformations are also characteristic of laterality defects. The candidate gene for CHD in this syndrome is *GATA4*, which maps to the 8p23.1 region and is expressed in the developing heart [38]. *GATA4* interacts with other transcriptional factors to drive DMP progression via SHH signaling [39].

### Deletion 3p25

Deletion 3p25 syndrome is also often associated with AVCD [40–42]. Cardiac malformations are diagnosed in about one-third of patients with deletion 3p25 patients [42]. In this syndrome AVCD is usually complete and *CRELD1* gene is the “critical “gene, based on its map position on chromosome 3p25 and considering that it is known to be causally related also to non-syndromic AVCD [43, 44]. The study of Burnicka-Turek et al. suggested that *CRELD1* mutations can cause AVCD acting on SHF Hh signalling [45].

### Syndromic AVCD and monogenic disorders

#### Ciliopathies

Several syndromes with AVCD are known to be pathogenetically related to ciliary dysfunction. This is not surprising considering that DMP development requires cilia-based Shh signaling. In fact, the role of Hedgehog signaling in coordinating multiple aspects of left-right lateralization and cardiovascular growth is well known. In addition, Sonic Hedgehog knock-out mice show CHDs in the setting of *heterotaxy* and left pulmonary isomerism [46–48].

Ciliopathies with AVCD can be divided in syndromes with polydactyly and syndromes without polydactyly. Among syndromes with polydactyly, ciliary dysfunction through abnormal processing of the Hh proteins has been documented in Ellis-van Creveld and other short-rib polydactyly, Smith-Lemli-Opitz, and oral-facial-digital type IV syndromes [22, 23, 49] while ciliary function is directly involved in Bardet-Biedl, oral-facial-digital I and VI syndromes [20, 21, 50, 51].

Syndromes with ciliary involvement and AVCD without polydactyly include VACTERL association and Alveolar Capillary Dysplasia.

AVCD in the context of these syndromes shows anatomical similarities with cardiac malformations found in *heterotaxy* and polysplenia [3, 52].

#### * Ellis-van Creveld syndrome

The Ellis-van Creveld syndrome is an autosomal recessive disorder characterized by short-limb dwarfism, short ribs, postaxial polydactyly of hands and feet, ectodermal defects and CHD [53]. Cardiac malformations are diagnosed in about two thirds of affected patients, prevalently AVCD associated with common atrium and systemic and pulmonary venous abnormalities [13, 52, 54]. Interestingly, AVCD is rarely associated with common atrium in the non-syndromic patients, but frequently associated in *heterotaxy* [55]. In the majority of the cases, Ellis-van Creveld syndrome is due to mutations in *EVC* and *EVC2* genes but mutations in *WDR35* and *DYNC2LI1* gene have been demonstrated in single patients. *EVC* and *EVC2* genes are required for normal transcriptional activation of Indian Hedgehog signalling [22, 53], with involvement of the proximal end of the primary cilium function [56]. The *WDR35* encodes a retrograde intraflagellar transport (IFT) protein that is required for the recruitment of the EVC-EVC2-SMOH complex to the cilium [57]. The *DYNC2LI1* gene codes for a component of the intraflaggelar transport-related dynein-2 complex, required for cilium assembly and function [58, 59].

#### * Oral-facial-digital syndromes

The oral-facial-digital syndromes include a group of 18 clinical subtypes with overlapping clinical features, including malformations of the face, oral cavity, and digits (polysyndactyly) [60]. CHD can also been present, and AVCD has been frequently diagnosed in patients with OFD syndrome type II [61] and type VI [62] and common atrium in OFD syndrome type I [63].

Several genes related to ciliary function and/or Sonic Hedgeghog signalling have been identified, as the X-linked dominant *OFD1* gene*,* encoding for a centrosomal protein involved in ciliary function [64], the *WDPCP* gene linked to the planar cell polarity ciliogenesis [65] and the *TCTN3* gene implicated in transduction of Sonic Hedgehog signalling [49].

#### * Joubert syndrome

Joubert syndrome is a group of genetically heterogeneous conditions characterized by multiorgan involvement (retinal, renal, hepatic and skeletal) and the pathognomonic neuroradiological “molar tooth sign”. Joubert syndromes can be associated with CHDs, including left ventricular obstructions, alone or associated with AVCD [52, 66]. Joubert syndromes are classified among ciliopathies, and more than 30 causative genes have been reported by now [67].

#### * Bardet-Biedl syndromes

Bardet-Biedl syndrome is an autosomal recessive disorder characterized by obesity, retinitis pigmentosa, postaxial polydactyly, genitourinary malformations, cognitive impairment, and CHD [68]. Laterality defects are described, including AVCD, dextrocardia without structural cardiac defects and abdominal *situs inversus* [23, 69, 70]. The AVCD can be considered the “classic” CHD in this syndrome. The syndrome is genetically heterogeneous, with several genes implicated, whose proteins are involved in ciliary function regulation [20].

#### * Smith-Lemli-Opitz syndrome

Smith-Lemli-Opitz syndrome (SLOS) is an autosomal recessive syndrome characterized by developmental delay, growth retardation, cleft palate, CHD, hypospadia, toe syndactyly, postaxial polydactyly, and facial anomalies [71]. CHD occurs in one-half of patients with SLOS [72]. Septal defects and AVCD are the most common CHDs and AVCD is often associated with anomalous pulmonary venous return, the latter being also a cardiac manifestation of *heterotaxy* with asplenia [72].

SLOS is due to an inborn error of cholesterol metabolism with deficiency of the 7-dehydrocholesterol-7 reductase (DHCR7) activity, due to mutations in the *DHCR7* gene. Cholesterol plays a critical role in formation of the normally active hedgehog proteins. Abnormal processing of Hedgehog proteins secondary to abnormal cholesterol levels seems to have a role in the development of SLO syndrome malformations [73].

#### * VACTERL association

VACTERL is a non-random association of congenital anomalies. Main clinical features are including vertebral defects (V), anal atresia (A), esophageal atresia (TE), radial and renal dysplasia (R) and limb anomalies (L), but CHDs are also an important finding in 50–80% of patients. Anatomic types of CHDs include septal, conotruncal and laterality defects (dextrocardia, *heterotaxy*, AVCD and transposition of the great arteries) [74].

The causal mechanisms underlying VACTERL association are heterogeneous and not completely established. Clinical observations and molecular studies in mice are showing that the association could be caused by defective SHH signaling and ciliopathies could be involved [75–77]. Genes described to cause the spectrum of malformations of VACTERL association include *Ift42* [78], *FOXF1* [77] and *ZIC3* [76, 77].

### Alveolar capillary dysplasia

Alveolar capillary dysplasia is a congenital pulmonary vascular abnormality, often associated with misalignment of the pulmonary vessels. The disease is associated with CHD in about 10% of the cases, prevalently consisting in partial or complete AVCD and various degrees of left heart obstruction (small left ventricle with or without aortic coarctation) [79].

Alveolar capillary dysplasia is caused by *FOXF1* gene mutations. Several studies demonstrated that *FOXF1* gene is activated by Sonic Hedgehog signaling [80].

### RASopathies

The term RASopathies includes the Noonan Syndrome and similar related syndromes (i.e., the LEOPARD syndrome or “Noonan syndrome with Multiple Lentigines”, the cardio-facio-cutaneous syndrome, the Costello syndrome, the Mazzanti syndrome and others) caused by mutations in genes encoding proteins with a role in the RAS/MAP kinase (MAPK) signalling pathway [81, 82].

The RASopathies are characterized by distinctive facial features, growth retardation, CHD, skeletal anomalies and variable neuropsychological deficits [81]. CHD occurs in about 65–85% of cases, depending on the mutated genes. Although pulmonary valve stenosis with dysplastic leaflets and hypertrophic cardiomyopathy of left ventricle are the most frequent cardiac defects, AVCD was also described. *PTPN11* and *RAF1* gene mutations have been prevalently detected in patients with AVCD associated with RASopathies [83–85]. AVCD is usually partial and may be associated with systemic obstructions including subaortic stenosis or aortic coarctation [85]. Structural abnormalities causing congenital subaortic stenosis include accessory fibrous tissue and/or anomalous insertion of mitral valve and anomalous papillary muscle of left ventricle [83–85].

Normal SHP2/PTPN11 function seems to act as IHH suppressor, and experiments in mice have documented decreased IHH levels in Noonan syndrome caused by germline activating mutations in *PTPN11* [86].

### CHARGE syndrome

CHARGE syndrome is characterized by ocular coloboma, choanal atresia, growth and developmental delay, genital anomalies and hearing loss. CHD is detectable in about 85% of patients with CHARGE syndrome [87] and AVCD is the second most frequent cardiac malformation, often in association with tetralogy of Fallot [88, 89].

The syndrome is caused by mutations in the *CHD7* gene in the majority of the patients [90].

### Holoprosencephaly

**Fig. 2:**
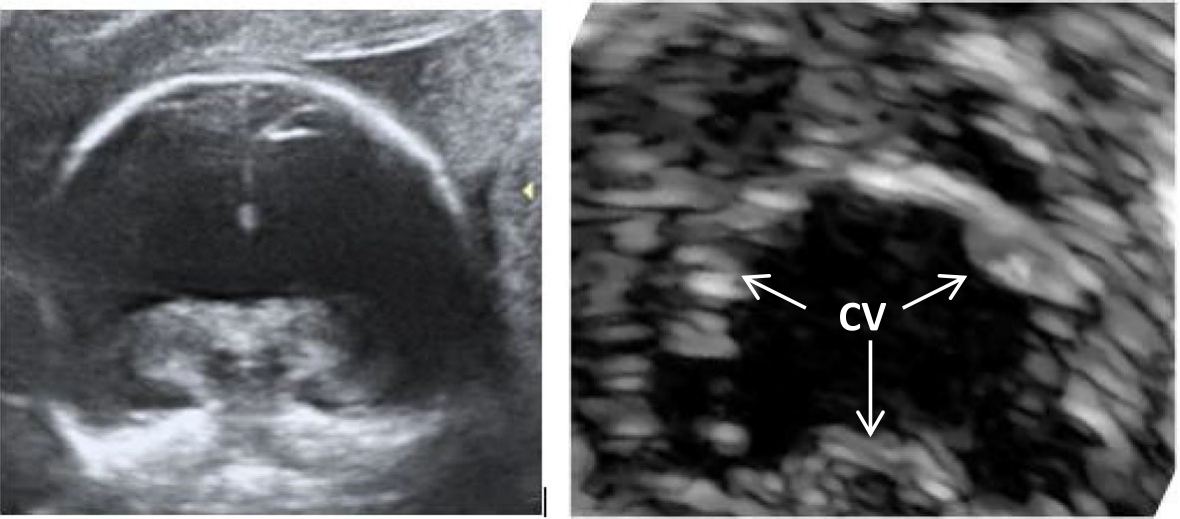
**a** Coronal sonogram of fetal head with alobar holoprosencephaly. **b** Echocardiographic subcostal view of common atrioventricular valve in the context of complete AVCD. CV: common valve

CHDs including septal defects have been described also in patients with holoprosencephaly [91]. Holoprosencephaly (HPE) is a severe congenital forebrain disorder usually associated with a broad spectrum of facial anomalies ranging from single axillary dental incisor and hypotelorism to extreme features such as cyclopia, proboscis and cleft lip with or without cleft palate. Shh role on commitment of the midline of neural structures is well known. Until now, at least 10 HPE loci have been identified (*Shh* [92, 93], *DKK1* [94],*GLI* [95], *SIX3* [96], *PTCH1* [97], *TDGF1* [98], *TGIF* [99] and *ZIC2* [100]). All the genes previously mentioned functionally interact or regulate the Shh concentration to drive forebrain development and ventral midline cell induction during different embryonic stages. In fact, the Shh −/−(null) mouse embryo displays a severe form of HPE [46, 92, 93, 101]. A correct regulation of Shh concentration is therefore crucial for the correct brain septation. However, Shh signaling pathway is deeply implicated also in ciliary function and acts on the DMP to drive the proper development of the cardiac AVC. In fact, in human beings, Shh pathway dysregulation has a well known impact on different types of AVCD [23]. This molecular considerations are supported by the striking phenotypical similarities between sonogram images of HPE (due to SHH deficiency in brain development) (Fig. 2a) and echocardiographic images of AVCD (Fig. 2b). Images (and phenotypes), indeed, support the unifying role of Sonic Hedgehog signalling on the commitment of midline structures of both brain and heart.

### Ethnic variations

In different ethic population AVCD can show distinct prevalence also in the context of the same syndrome supporting the multiple genetic origin of this CHD. In particular, in the context of DS, several studies highlight the effect of sex and ethnic factors in addition to trisomy 21 to determine different prevalence of AVCD.

It is notable that in oriental and native-American DS patients the most frequent CHD is represented by VSD whereas in Caucasian DS populations AVCD are prevalent [102–104]. Freeman et al. reported significant ethnic differences in the prevalence of AVCD in DS patients. The study demonstrated that blacks with DS were twice as likely to be born with a complete AVCD whereas Hispanics DS patients showed a trend toward fewer AVCD [105].

### Non-syndromic atrioventricular canal defects

The majority of AVCD not related to trisomy 21 occur as sporadic cases [13] and non-syndromic patients with visceroatrial *situs solitus* (without *heterotaxy*) account for about 25% [13]. Indeed, AVCD prevalence decreases to 0.97–1.32 per 10,000 livebirths looking at non-syndromic cases (Fig. 1). In this population of non-syndromic patients, only 3–5% show familial recurrence. The autosomal dominant pattern of inheritance is prevalently involved, sometimes with incomplete penetrance. There is emerging evidence that maternal risk factors (genetic and environmental) can confer a major risk for non-syndromic CHD [106, 107].

The first gene mapped for AVCD was *CRELD1*, located inside the “CHD critical region” on 3p25, known as the AVCD2 locus. *CRELD1* gene acts as a regulator of calcineurin/NFATc1 signaling which is crucial for the regulation of cardiac development. In fact, NFATc signaling determines valve initiation and maturation, regulating the activity of VEGF to undergo endomesenchimal transition (EndoMT) [108]. *CRELD1* the most frequently AVCD associated gene, since heterozygous mutations have been shown to occur in about 6% of non-syndromic partial AVCD [109]. In addition, some *CRELD1* gene mutations, including the c.985C > T (p.Arg329Cys) as recurrent one [110], have been reported to be a risk factor for CHD also in patients with DS [111]. Experimental studies in mice have shown that the introduction of a null allele of *Creld1* in theDs65Dn mouse can increase the prevalence of CHDs [112]. Interestingly, a link between CRELD1 and ciliary dysfunction through disruption of Shh signaling has been suspected [45, 113].

The fact that defective NFATC1 function could contribute to isolated AVCD was also demonstrated by a recent work by Ferese et al. [114]. The authors reported missense rare variants in *NFATC1* gene in two patients with non-syndromic AVCD and in one syndromic patient with AVCD in the context of heterotaxia and polysplenia with left isomerism. Experimental studies in zebrafish have demonstrated that NFATC1 variants have a great impact on cardiogenesis, affecting specifically cardiac looping process. Interestingly, a link between *NFATC1* and *CRELD1* genes has been noted, since *CRELD1* has been shown to be a master regulator of calcineurin/NFATC1 signaling [114].

Several studies highlight the importance of testing “syndromic” genes when investigating patients with isolated CHDs. Some genes causative or contributory for specific syndromes with cardiac involvement can play a role also in isolated AVCD. In fact, linkage studies of familial AVCD first excluded chromosome 21 loci in the pathogenesis of isolated sporadic AVCD [115, 116].

Weissman et al. [117] reported a non-synonymous mutation of *PTPN11* in a subject with isolated complete AVCD. Missense mutation of this gene account for approximately 50% of Noonan syndrome, an autosomal dominant disorder presenting with atrioventricular septal defects in almost 15% of cases.

Recently, D’Alessandro et al. [118] performed a NGS (exome sequencing) analysis in a large cohort of unrelated AVCD probands and in a replication cohort of unrelated, non-syndromic, Caucasian AVCD probands. Data for replication analysis were obtained from population databases. The authors found rare damaging non-synonymous variants in six genes (*NIPBL*, *CHD7*, *CEP152*, *BMPR1a*, *ZFPM2*, *MDM4*) all known for their association with some syndromes with CHDs. In humans there is a considerable phenotypic heterogeneity in AVCD whereby different genes can contribute to the same phenotype. For these reasons, NGS is a powerful tool that has the potential to increase the specificity and accuracy of the observed results.

One of the most robustly CHD-associated gene is *GATA4*, mapping on the “CHD critical region” 8p23.1 [38]. *GATA4* is a developmental transcription factor associated with atrial septal defects and ventricular septal defects but also with non-syndromic AVCD.

*GATA4* is required for proliferation of SHF atrial septum progenitors and for the progression of the DMP via Hedgehog signaling. The role of *GATA4* in cardiac AVC septation is therefore deeply dependent on Shh signaling [39].

Thanks to the wide spread of NGS techniques additional locus for isolated AVCD have been found. Rare de novo missense variants in NR2F2 were described by Al Turky et al. in 13 trios and 112 unrelated individuals with non-syndromic AVCD [119]. The role of NR2F2 gene on cardiogenesis was postulated on the basis of a previously published mutant mouse that shows defective endothelial mesenchymal transformation and hypocellularity of the atrioventricular canal, strongly suggesting a role for NR2F2 in cardiac developmental in a dosage-sensitive fashion [120].

Priest et al. in a recent study confirmed that de novo mutations may account for a small fraction of isolated CHDs [121]. The authors found rare de novo variants in multiple genes (NR1D2, ADAM17, RYR1, CHRD, PTPRJ, IFT140, ATE1, NOTCH1, NSD1, ZFPM2, MYH6, VCAN, SRCAP, KMT2D, NOTCH2, BBS2, EHMT1) surveying a multi-institutional cohort, combining analysis of 987 individuals (discovery cohort of 59 affected trios and 59 control trios, and a replication cohort of 100 affected singletons and 533 unaffected singletons). The study was ruled out combining both exome-sequencing and array-CGH, suggesting a locus heterogeneity and a oligogenic inheritance of isolated AVCD.

The possible role of genomic structural variants such as copy number variants (CNV) in the etiology of non-syndromic AVCD has only been studied in a minority of cases. Priest et al. [122] identified two sub-chromosomal deletions occurring in cr20p12.3 and in cr3q26.1 respectively, previously not directly linked to AVCD. However, the deletions found at these loci contain some genes that can be linked to cardiac morphogenesis. The authors, indeed, conclude that large CNV might confer a minor risk for isolated AVCD.

The studies cited above indicate that isolated non-syndromic AVCD is a highly genetically heterogeneous malformation that probably requires an unknown combination of factors to break the theoretical disease threshold. Noteworthy, specific genes implicated in different steps of cardiogenesis can have a contributory role in different CHD. This observation provides additional evidence of the wide molecular heterogeneity in establishing cardiac phenotype and highlights the fact that CHDs are not to be considered monogenic disorders.

### Familial AVCD

The Baltimore Washington Infants Study revealed that among non-syndromic children showing CHDs, only 3–5% presented familial recurrence. Studies on several pedigrees showed that the recurrence risk for CHD among siblings of patients with AVCD was about 3.6% [123], similarly to the mean recurrence risk reported in previous studies [124].

Traditionally, segregation analysis in families with AVCD suggested an autosomal dominant pattern of inheritance related to a major gene. The hypothesis that AVCD shared a monogenic or oligogenic pattern of inheritance agreed with the clinical observation that CHDs in the offsprings were concordant with cardiac defects in parents [123]. Nevertheless, recent studies on large pedigrees highlight low concordance ratios in families and importance of sex and ethnical drive as risk factor for recurrence rates. These observations support the multigenic origin of familial AVCD that often shows complex traits of inheritance with incomplete penetrance [125, 126].

Molecular basis of familial AVCD are largely unknown. Due to the fact that AVCD represent the major CHD among DS patients, candidate genes on chromosome 21 were firstly investigated with linkage analysis studies. The results, however, excluded the involvement of chromosome 21 “critical region” loci [115, 116, 127]. Exclusion of linkage with chromosome 21 in families with recurrence of non-syndromic AVCD was also consistent with previous observations on anatomic differences between Down and non-Down AVCD [13].

Some genes deeply implicated in cardiogenesis have been found in pedigrees with AVCD. Missense mutation in CRELD1 gene, mapping on cr3p, has been described in the context of familial AVCD [128, 129] as well as mutations in *PTPN11* [117],*GATA4* [130] and the *p93* gene, mapping on chromosome 1 p [131].

A recent work of Demal et al. reported a family with multiple cardiac defects including AVCD and found out that every affected family member carries a BMPR1A missense mutation. *BMPR1A* is required to ensure the correct development of endocardial cushions via EndoMT regulating the Wnt/ß-catenin signalling. The reported BMPR1A variant leads to reduced atrioventricular valve area and ectopic valvular tissue in experimental studies in zebrafish and is to be considered a potential candidate gene in the development of non-syndromic AVCD [132].

Familial and isolated cases of AVCD sometimes show variants in genes encoding for transcriptional factors deeply implicated in cardiogenesis such as *TBX20* and *Tbx2*. *Tbx20* is a T-box transcription factor that interacts with *Tbx2* to promote EndoMT and proliferation of the AVC tissue. Therefore this gene directly acts on endocardial cushion formation [133].

Mutations in well known genes account only for a small percentage of familial AVCD, whereas the majority of isolated AVCD with familial recurrence seems to have a complex etiology based on a variety of genes. Combination of traditional linkage analysis techniques with genome and exome sequencing represent a powerful tool to evaluate complex trait of recurrence of this CHDs.

A better understanding of the molecular basis of familial AVCD could have a significant impact on clinical outcome driving a correct genetic counseling based on a focused family history.

### Implications for clinical practice

The knowledge of genetic basis of AVCD can be useful for prenatal and postnatal clinical management of affected patients.

Information about the prevalence and type of genetic syndromes possibly associated with AVCD can be useful for clinicians involved in prenatal controls and for targeted screening for extracardiac defects. The link between anatomic types of AVCD and specific genetic syndrome could be a marker in diagnostic work. The large genetic heterogeneity of AVCD associated with the possible limits of prenatal genetic testing should be known in prenatal counseling.

In postnatal management of syndromic patients with AVCD it is important to try to perform an early and precise genetic diagnosis. This can lead to knowledge of risk factors, early monitoring and treatment of extracardiac defects, the use of specific multidisciplinary protocols and guidelines.

Genetic counseling to families is also important. Molecular diagnosis in the proband gives the possibility to test the parents and other relatives, in order to precise the possible familial genetic risk. Based on the present genetic knowledge, the molecular approach is more suitable for syndromic rather than non-syndromic AVCD.

## Conclusions

**Fig. 3:**
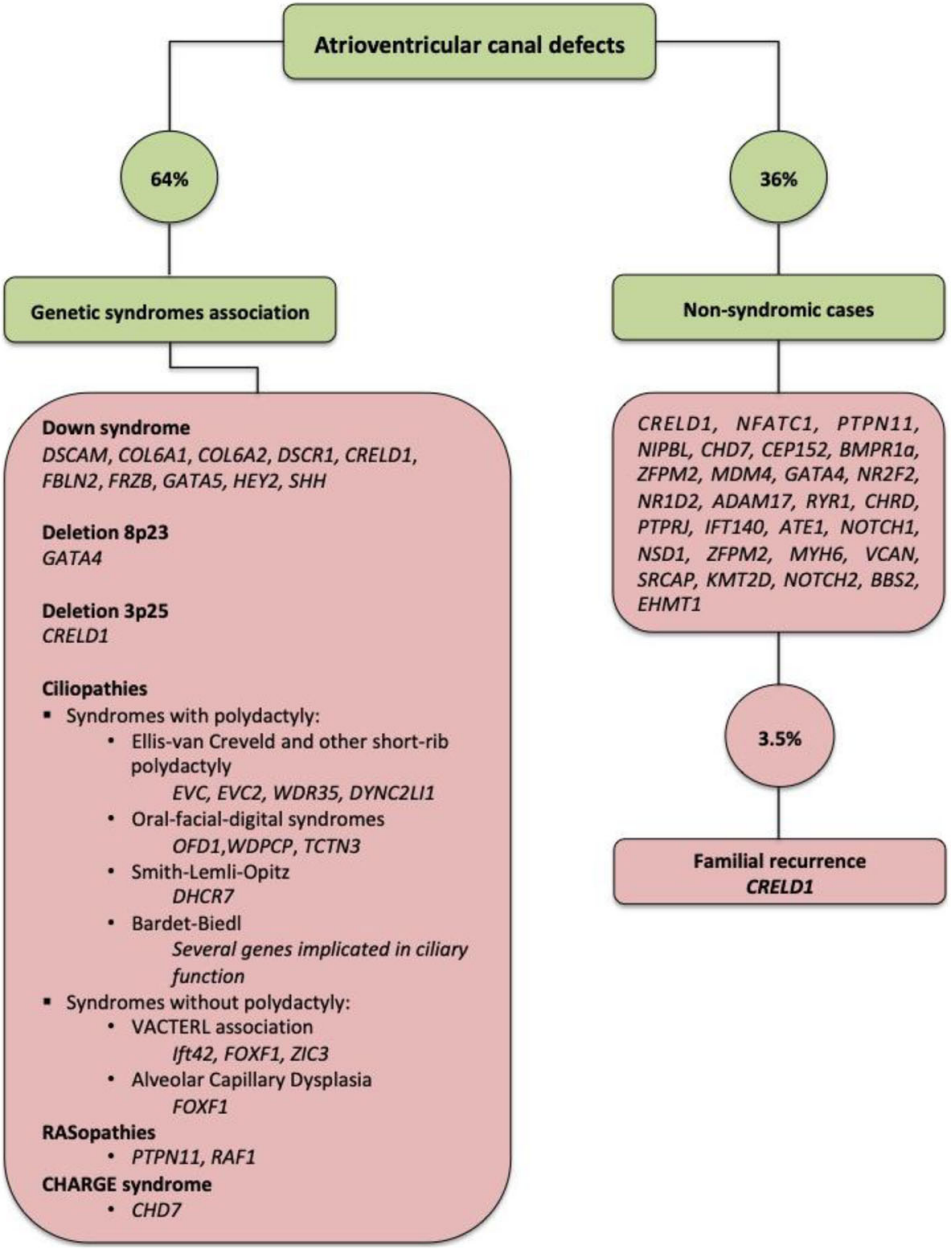
Genes involved in different forms of atrioventricular canal defects

AVCD is a very heterogeneous cardiac phenotype that frequently occurs in association with several genetic syndromes. A better understanding of AVCD molecular background could have relevance in different clinical settings. As cited above, AVCD knowledge could drive proper genetic counselling increasing clinical usefulness of fast and high resolution tools for *prenatal diagnosis* such as array-CGH platforms (Fig. 3). Anatomic differences in AVCD can be caused by distinct genetic diseases. Nevertheless, molecular studies are demonstrating that several genes responsible for syndromes with AVCD can be involved in ciliary function and/or abnormal processing of proteins implicated in Hedgehog signaling. Anomalies in different components of the Hedgehog pathway can express in syndromic AVCD associated with partially overlapping clinical extracardiac manifestations.

Several studies indicate a complex genetic trait involved in non-syndromic ACVD and highlight that the physiopathology of isolated AVCD depends on multiple molecular mechanisms.

During early cardiogenesis the correct specification of the atrial and ventricular chambers relies on two equally important embryogenetic processes. On one hand the primary intracardiac mechanism driven by the maturation of endocardial cushions via EndoMT and, on the other hand, the extracardiac mechanisms led by activation of DMP via Shh signalling to complete the AVC septation [23].

Although the pathogenesis of syndromic AVCD seems to be deeply related to DMP development driven by Shh signaling, probably in isolated non-syndromic AVCD the primary embryological step of endocardial cushion tissue proliferation following EndoMT should be still considered as an important pathogenetic mechanism.

The pathogenesis of both syndromic and isolated AVCD, however, appears to be as complex as still not completely understood. Targeted NGS offers a great opportunity to improve sensibility and specifity of genetic analysis for AVCD.

Similarly to conotruncal heart defects in the context of 22q11.2 deletion syndrome and branchial arch anomalies, AVCD can be considered as a phenotypic marker linking all syndromes related to cilia through Shh pathway. Hence, we postulate that AVCD should be considered as part of “developmental field” as introduced by Opitz et al. [134, 135].

## Data Availability

The data that support the findings of this study are available from the corresponding author upon reasonable request.

## Abbreviations

AVCD: Atrioventricular canal defect
CHD: Congenital heart defect
NGS: Next Generation Sequencing
DS: Down syndrome
DMP: Dorsal mesenchymal protrusion
SHF: Secondary heart field
IFT: Intraflagellar transport
SLOS: Smith-Lemli-Opitz syndrome
HPE: Holoprosencephaly
SHH: Sonic Hedgehog
EndoMT: Endomesenchimal transition
CNV: Copy number variants
